# Study on the Axial Compressive Behavior and Constitutive Relationship of Lightweight Mixed Ceramic Concrete

**DOI:** 10.3390/ma18020390

**Published:** 2025-01-16

**Authors:** Yanxia Huang, Weiying Huang, Qunyi Huang, Wanyong Tuo, Qingchao Feng

**Affiliations:** 1 School of Civil and Transportation Engineering, Anyang Institute of Technology, Anyang 455000, China; huangyanxia0301@outlook.com (Y.H.); twy2014@163.com (W.T.); 2 School of Civil Engineering, Southwest Jiaotong University, Chengdu 610031, China; hqy1986@swjtu.edu.cn; 3 Henan Yuanda Sustainable Building Technology Co., Ltd., Anyang 455000, China; fqc2025@163.com

**Keywords:** lightweight mixed ceramic concrete, axial compressive strength test, stress–strain relationship, mechanical behavior

## Abstract

To thoroughly study the stress–strain relationship of lightweight mixed ceramic concrete, this paper conducts axial compressive strength tests on three groups of lightweight mixed ceramic concrete specimens with different types and contents as the basis. It establishes the elastic modulus calculation formula and compressive stress–strain formula for lightweight mixed ceramic concrete by combining with the current standards and related research. The results show that lightweight mixed ceramic concrete, made of a mixture of different types and densities of ceramic grains, has better mechanical properties and deformation properties. The calculation errors of the modulus of elasticity formulas, derived from the experimental results for the three groups of lightweight mixed ceramic concretes, are all controlled within 5%. The average relative errors of the fitting results of stress–strain curves for the three groups of specimens and the measured data are as low as 6.66%, 3.16%, and 3.39%. The errors between the experimental values of the modulus of elasticity of different studies and the predicted values based on the formula in this paper were controlled within 17%, and the average relative errors between the predicted and experimental results of the stress–strain curves for the three groups of specimens were 2.64%, 8.94%, and 17.50%. This paper innovatively constructs a prediction model of key mechanical parameters of lightweight mixed ceramic concrete, which can provide a reference and experimental basis for the structural analysis and application of lightweight mixed ceramic concrete.

## 1. Introduction

Lightweight ceramic concrete is a kind of concrete with a dry apparent density lower than 1950 kg/m^3^. It has the advantages of good heat preservation, seismic, seepage, and frost resistance, and has gradually become a hot spot in the construction industry [1,2]. The stress–strain relationship of concrete under axial compression is one of the basic datasets for studying the mechanical properties of concrete materials. It is also an important basis for researching and establishing the analysis of bearing capacity, deformation, and the whole process of stressing concrete members. However, lightweight ceramic concrete is affected by the characteristics of the ceramic grains themselves, causing a significant difference in the material’s mechanical properties compared to traditional concrete. In the in-depth study of lightweight ceramic concrete, the direct selection of traditional concrete stress–strain relationship data will seriously reduce the accuracy of the research results [3,4]. Therefore, it is of great significance to investigate the stress–strain relationship of lightweight ceramic concrete and construct a reasonable constitutive equation for its further application and promotion in the construction field.

In the study of constitutive equations for lightweight ceramic concrete, the researchers conducted numerous experimental studies based on the differences in coarse aggregates, yielding remarkable results [5,6,7]. Currently, lightweight ceramic concrete primarily uses shale ceramsite or fly ash ceramsite as its coarse aggregate [8]. However, single-type ceramic concrete often faces several issues, including low compressive strength, brittleness, susceptibility to cracking, overall in-homogeneity, and instability [9,10]. Based on this, Hou et al. [11], Wei et al. [12], and Yang et al. [13] concluded that lightweight mixed ceramic concrete (LWMCC) can effectively enhance the mechanical, deformation, and work properties of lightweight ceramic concrete. Although these studies have mostly focused on using experimental methods to investigate how ceramic mixtures affect the strength, modulus of elasticity, and deformation of LWMCC, they have not yet discovered a physical characteristic that can describe the kind and quantity of ceramsite. Such a parameter would reflect their impact on the mechanical properties of LWMCC, including strength, modulus of elasticity, and the stress–strain relationship.

Considering that the density of ceramsite can comprehensively reflect the type and content of ceramsite and is closely related to the mechanical properties of ceramic concrete [14,15], this paper is based on the axial compressive performance test of LWMCC and introduces the density of ceramsite as a key parameter to modify the elastic modulus and constitutive relationship model of LWMCC. The purpose of this paper is to propose a stress–strain relationship model suitable for LWMCC with different types and contents of ceramsite, in order to provide theoretical support and data reference for the engineering application of LWMCC, thereby promoting its further application and promotion in the construction field.

## 2. Lightweight Mixed Ceramic Concrete Test

### 2.1. Test Materials and Loading Systems

The types of ceramic grains selected for the test were shale ceramic grains and fly ash ceramic grains. All the ceramic grains were soaked in advance for 3 h. In accordance with Chinese standard GB/T 17431.2-2010 [16], the barrel compressive strength, bulk density, and water absorption of the ceramic particles were measured. Additionally, the modulus of elasticity for the ceramsite was calculated based on the referenced literature [17], with specific parameters detailed in Table 1. The sand was mechanism sand with a bulk density of 1400 kg/m^3^; the cement was P.O42.5R grade ordinary silicate cement. The cement was P.O42.5R grade ordinary silicate cement.

The loading device utilized in this study was a TSH206A universal testing machine, manufactured by Shenzhen Wance Test Equipment Co., Ltd., based in Shenzhen, China. The machine was tested in accordance with the Chinese standard GB/T50081-2019 [18]. Compressive strength values were obtained by the TSH206A universal testing machine. Strain values were obtained by testing with strain gauges. Strain gauges are applied in the vertical direction before and after the specimens, and the average values of the strain data obtained from these measurements are utilized as representative strain values for analysis.

### 2.2. Design of Mix Proportion

In this study, the orthogonal method was employed to standardize the quantities of cement, sand, water, and the volumetric ratio of ceramsite. The specimens were systematically categorized into three distinct groups, designated as A, B, and C, based on the type and content of the ceramsite utilized. The loose volume method [19] was employed to ascertain the mixing ratios for each group of LWMCC, as detailed in Table 2. Based on these ratios, four prisms measuring 100 mm × 100 mm × 300 mm were prepared for each group, totaling twelve specimens. The specimens were demolded after 24 h and were cured in the standard curing room for 28 days. This was carried out to examine the impact of different types of ceramsites on the mechanical properties of the LWMCC.

## 3. Experimental Results and Discussion

### 3.1. Axial Compressive Stress–Strain Curve

**Figure 1:**
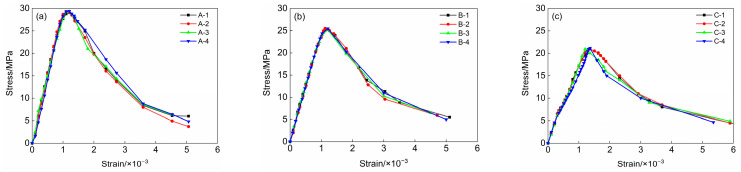
Axial compressive stress–strain curves of three types of LWMCC. (**a**) Axial compressive stress–strain curves of specimen A; (**b**) Axial compressive stress–strain curves of specimen B; (**c**) Axial compressive stress–strain curves of specimen C.

Figure 1 presents the stress–strain relationship curves for three distinct types of LWMCC prismatic specimens subjected to axial compression. The stress–strain relationship curves of specimens with the same mixing ratio show minimal differences in the ascending section; however, they exhibit more significant variations in the descending section. This indicates that LWMCC tends to be more brittle, with greater dispersion observed in the descending phase after reaching the peak value.

**Figure 2:**
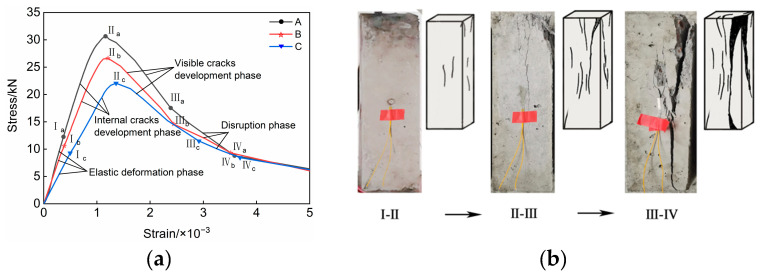
Load–displacement curves and characteristic points of prismatic specimens for axial compressive strength tests. (**a**) Stress–strain curve of prismatic specimens; (**b**) Cracking process of prismatic specimen B.

The average stress–strain relationship curves of three types of LWMCC specimens, labeled A, B, and C, are shown in Figure 2a. For the three distinct types of LWMCC specimens, the stress–strain relationship curves were derived by computing the average values for each category, as illustrated in Figure 2a. The damage progression of the specimens can be categorized into three distinct stages:

Initial Elasticity Stage (0–I): During this phase, the specimen exhibits no discernible cracks on its surface, and the relationship between stress and strain remains predominantly linear. This behavior indicates that the specimen is undergoing elastic deformation.

Elastic–plastic to the eve of destruction stage (I–II): As displacement increases, the specimen enters the elastic–plastic stage, characterized by the gradual emergence of fine cracks at both the top and central regions, accompanied by noticeable splitting sounds. These cracks continue to propagate with increasing loading displacement until one or more through-cracks, parallel to the loading direction, develop at the top of the specimen, ultimately reaching their maximum bearing capacity.

Post-destruction stage (II–IV): Once the specimen reaches its damage threshold, internal cracks begin to expand rapidly, ultimately resulting in the failure of the overall structure. During this stage, the specimen fractures into several smaller columns, displaying clear signs of brittle damage. Figure 2b illustrates the progression of cracks in the specimen at each stage, from initial loading to complete damage, using specimen B as a case study.

### 3.2. Characterization Indicator Analysis

#### 3.2.1. Peak Strain and Compressive Strength

**Figure 3:**
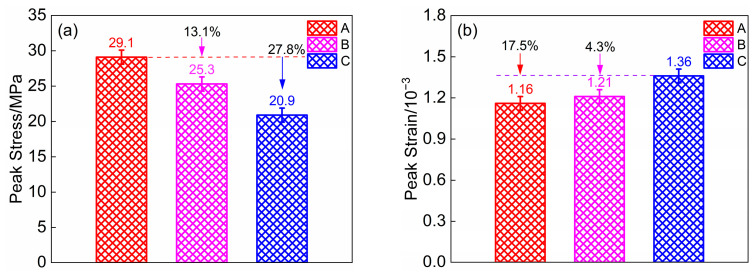
Comparison of peak stress and strain of three types of specimens. (**a**) peak stress of specimens; (**b**) peak strain of specimens.

Based on the axial compressive test data of the specimens, the peak stress and peak strain comparisons of the three groups of specimens were plotted separately, as shown in Figure 3.

As illustrated in Figure 3a, specimen A exhibits the highest compressive strength, which is 13.1% greater than that of specimen B and 27.8% higher than that of specimen C. This discrepancy indicates a negative correlation between the content of fly ash ceramsite and the compressive strength of the concrete; specifically, the higher the content of fly ash ceramsite, the lower the compressive strength of the specimen. This phenomenon can be attributed to the fact that LWMCC primarily experiences aggregate damage. As the compressive strength of the cylinder decreases, the content of ceramsite increases, resulting in diminished compressive strength of the LWMCC.

As shown in Figure 3b, the peak strain of specimen C is the highest, measuring 17.5%, which is 4.3% greater than that of specimens A and B, respectively. This suggests that while fly ash ceramsite impacts the strength of the specimens, it also enhances their deformation properties.

This is mainly because the enhanced moisture retention capabilities of high-water absorption fly ash ceramsite significantly mitigate self-shrinkage in LWMCC. This prolonged moisture availability not only minimizes the formation of cracks but also facilitates improved deformation properties in the ceramic concrete matrix. Moreover, the combination of pebble-type fly ash ceramsite with crushed stone-type shale ceramsite enhances aggregate densification. When cracks propagate through the ceramsite and damage the aggregate, or when damage occurs along the cement interface, the mixed ceramic concrete is able to dissipate more energy. This improvement contributes to the cracking resistance and deformation capacity of LWMCC.

When combined with Table 2 and Figure 3, it is evident that the compressive strength of Specimen B significantly exceeded that of Specimen C. Additionally, the peak strain of Specimen B was notably greater than that of Specimen A. These findings indicate that incorporating shale ceramsite into fly ash ceramsite enhances the mechanical properties of the ceramic concrete, while the addition of fly ash ceramsite to the shale ceramsite improves the deformation characteristics of the concrete. In summary, the synergistic combination of fly ash ceramsite and shale ceramsite significantly enhances the strength and deformation capacity of LWMCC, while concurrently preserving its lightweight properties.

#### 3.2.2. Elastic Modulus

**Figure 4:**
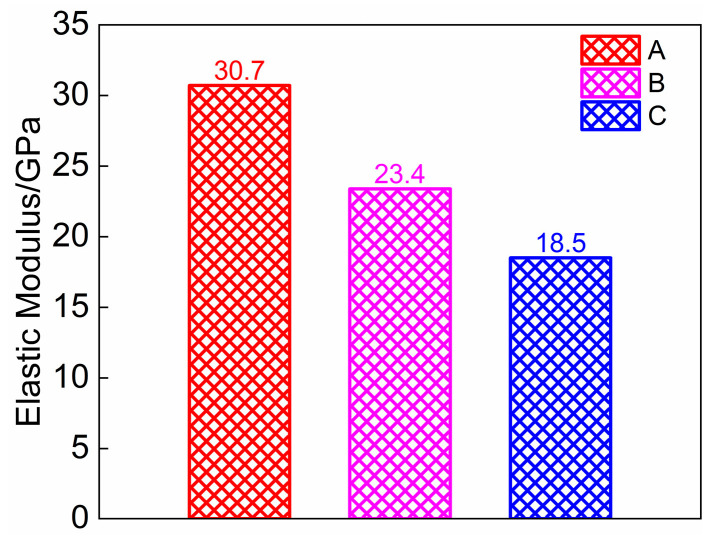
Elastic modulus of three types of specimens.

The modulus of elasticity for this test was determined by taking the ratio of the 1/3 axial compressive strength value of LWMCC to the corresponding strain value. The modulus of elasticity *E*_c_ of the three sets of specimens is shown in Figure 4. The analysis reveals that the modulus of elasticity for specimens A, B, and C decreases in a sequential manner. This indicates a negative correlation between the modulus of elasticity and the content of fly ash ceramsite; specifically, as the content of fly ash ceramsite increases, the modulus of elasticity of the specimens decreases. Conversely, a higher content of shale ceramic concrete results in an increase in the modulus of elasticity of the specimens.

The density of ceramic grains can comprehensively respond to the content of various ceramsites within a ceramic mixture. Consequently, this paper employs a density compensation method to refine the calculation of the Chinese standard GB 50010-2010 concerning the modulus of elasticity of concrete, as illustrated in Equations (1) and (2). The proposed formula considers the influence of various types and contents of ceramic grains on the modulus of elasticity. However, due to the high-density grade of ceramic grains utilized in the experiments, in this case, the expression is applicable when $\rho_{a}$ ≥ 566 kg/m^3^.

(1) $$E_{c}=\frac{10^{5}}{2.2+(34.7/f_{\mathrm{cu},k})}\times (3.36\rho_{a}-1.9)$$

(2) $$\rho_{a}=\frac{V_{f}}{V_{a}}\rho_{f}+\frac{V_{y}}{V_{a}}\rho_{y}$$

where $f_{\mathrm{cu},k}$ represents the standard value of cubic compressive strength of LWMCC; $\rho_{a}$ represents the density of mixed ceramsites, measured in g/cm^3^; $\rho_{f}$ represents the density of fly ash aggregate particles; $V_{f}$ represents the volume of fly ash aggregate particles; $\rho_{y}$ represents the density of shale ceramsite; $V_{y}$ represents the volume of shale ceramsite; $V_{a}$ represents the volume of mixed ceramsites.

The modulus of elasticity is determined using Equations (1) and (2), with the associated errors from the test results presented in Table 3. It is evident that the error in the modulus of elasticity for the specimens from groups A, B, and C has been limited to 5%. This indicates that the elasticity modulus formula proposed in this paper can effectively calculate the modulus of elasticity for LWMCC.

## 4. Stress–Strain Equations and Validation

### 4.1. Presentation of the Stress–Strain Equation

The existing literature identifies three primary methodologies for the calculation of compressed concrete. In Table 4, the mathematical formulations corresponding to these three models are delineated, specifically focusing on the ascending and descending segments of the stress–strain curve.

To assess the accuracy of the three models predicting the stress–strain relationship of LWMCC, we employ the coefficient of determination and the average relative error, as outlined in Equations (3) and (4). The coefficient of determination, *R*^2^, serves as a comprehensive metric for evaluating the model’s goodness of fit to the sample data; a higher *R*^2^ indicates a more effective fit. In contrast, the average relative error (*AARE*) reflects the accuracy of the predicted results—with a lower average error value denoting greater prediction accuracy.

(3) $$R^{2}=\left(\sum_{i=1}^{n}{\left(C_{i}-\bar{E}\right)}^{2}\right)/\left(\sum_{i=1}^{n}{\left(E_{i}-\bar{E}\right)}^{2}\right)$$

(4) $$AARE=\frac{1}{n}\sum_{i=1}^{n}\left|\frac{E_{i}-C_{i}}{E_{i}}\right|$$

where *E*_i_ represents the experimental data, measured in GPa; $\bar{E}$ represents the average of the experimental data; *C*_i_ represents the calculated data; *n* represents the number of calculation points.

**Figure 5:**
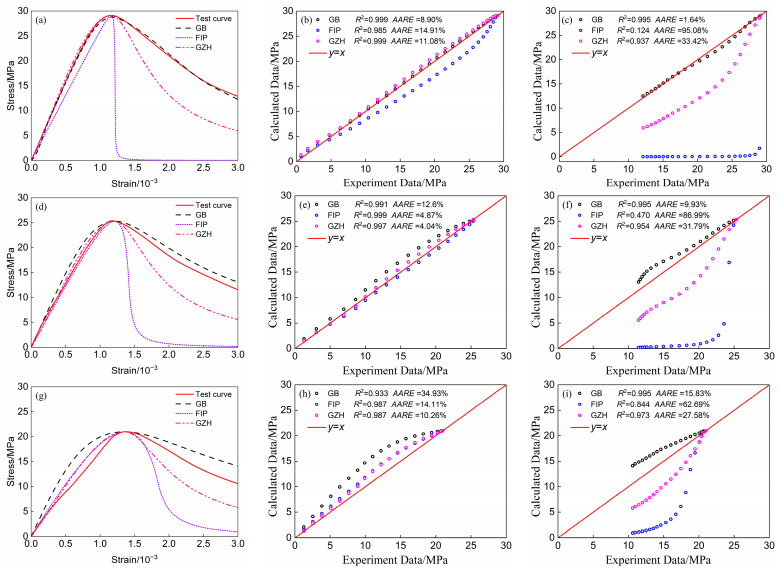
Comparison of the calculation results of the three models and the experimental analysis diagrams. (**a**) A stress–strain curve; (**b**) Analysis of the fit of the A rising segment; (**c**) Analysis of the fit of the A descending segment; (**d**) B stress–strain curve; (**e**) Analysis of the fit of the B rising segment; (**f**) Analysis of the fit of the B descending segment; (**g**) C stress–strain curve; (**h**) Analysis of the fit of the C rising segment; (**i**) Analysis of the fit of the C descending segment.

To assess the goodness of fit and prediction accuracy of the three computational models, 20 points were selected at equal intervals for both the ascending and descending segments, with the peak strain serving as the cut-off point. *R*^2^ and *AARE* were then calculated, and the results are presented in Figure 5.

As can be seen in Figure 5, in the ascending section, the dispersion points of the three calculation models are basically distributed around *y* = *x*. Additionally, the decidability coefficients for all models exceed 0.9, indicating a strong fit to the test curve. The GB model showcases a high level of computational accuracy specifically for specimen type A. In contrast, the FIP model demonstrates enhanced computational accuracy exclusively for specimen type B. Meanwhile, the GZH model attains relatively high computational accuracy across all three specimen types—A, B, and C. In the descending section, the FIP model proves unsuitable for the concrete specimens discussed in this paper. The computational errors of the GZH model for specimens B and C have significantly surpassed 10%, failing to meet the accuracy standards required in engineering. Conversely, the GB model demonstrates high accuracy for both specimens A and B, though the error for specimen C is 15.83%.

Since the GB model, the FIP model, and the GZH model are mainly designed for normal concrete, the existing models for the constitutive relationship of concrete appear to be inadequately suited for LWMCC, which incorporates varying proportions of ceramic materials. To develop a more refined ontological relationship model for LWMCC, it is essential to comprehensively assess the impact of varying ceramic content on the elastic modulus and related parameters of the concrete. In this paper, we introduce density as a key parameter closely linked to ceramic grain content. We also revise the existing ontological relationship model by incorporating elements from the GZH model for the ascending section and the GB model for the descending section, as illustrated in Equation (5).

(5) $$\sigma /\sigma_{c}=\left\{\begin{array}{cc} b{\left(\varepsilon /\varepsilon_{c}\right)}^{3}+c{\left(\varepsilon /\varepsilon_{c}\right)}^{2}+d\varepsilon /\varepsilon_{c} & \varepsilon \leq \varepsilon_{c}\\ \frac{\varepsilon /\varepsilon_{c}}{g{\left(\varepsilon /\varepsilon_{c}\right)}^{2}+h\varepsilon /\varepsilon_{c}+i} & \varepsilon_{c}\leq \varepsilon \end{array}\right.$$

where *b*, *c*, *d* represent the parameter for ascending section; *g*, *h*, *i* represent the parameter for descending section, all relate to density; $\varepsilon_{c}$ parameters peak pressure strain for prisms; $\sigma_{c}$ parameters peak pressure stress for prisms.

**Figure 6:**
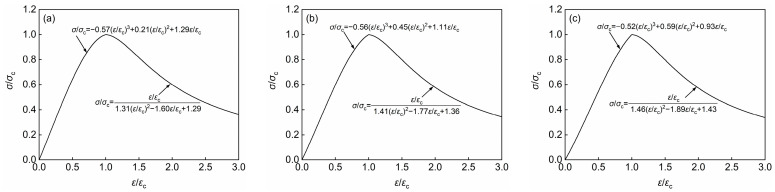
Normalization fitting curves of three types of LWMCC. (**a**) Normalized fitting curve for Group A specimens; (**b**) Normalized fitting curve for Group B specimens; (**c**) Normalized fitting curve for Group C specimens.

The LWMCC test data were normalized and fitted. The outcomes of these analyses are illustrated in Figure 6.

**Figure 7:**
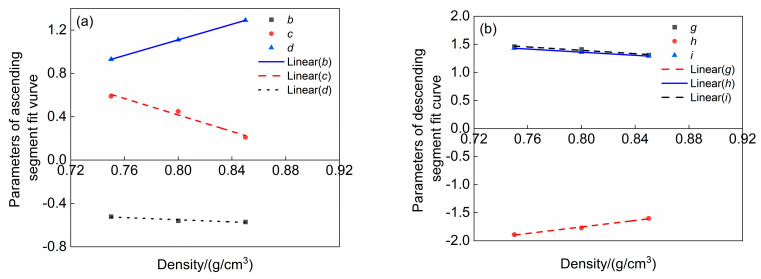
Parametric fitting of stress–strain curves for three types of LWMCC. (**a**) Parametric fitting of ascending section; (**b**) Parametric fitting of descending section.

Based on the stress–strain normalized fitting curves of three types of LWMCC presented in Figure 6, this paper examines the relationship between aggregate density and key parameters in the stress–strain normalized fitting curves of LWMCC using regression analysis. It identifies the relationships between aggregate density and the key parameters *b*, *c*, and *d* in the ascending section, as well as *g*, *h*, and *i* in the descending section, which are illustrated in Figure 7. As the aggregate density increases, a corresponding increase in the parameters d and h is observed, while parameters *b*, *c*, and *i* exhibit a decreasing trend. This relationship suggests that a rise in aggregate density enhances the plasticity of LWMCC.

So we establish that b=−0.5ρ−0.15, c=3.46−3.8ρ, d=3.6ρ−1.77, g=2.59−1.5ρ, h=2.52−1.4ρ, i=2.9ρ−4.07. By substituting these six parameters into Equation (5), one can derive a model that elucidates the intrinsic relationship of LWMCC, as described by Equation (6). In alignment with the constraints in the modulus of elasticity formula, the stress–strain relationship formula is applicable when $\rho_{a}$ ≥ 566 kg/m^3^.

(6) $$\sigma /\sigma_{c}=\left\{\begin{array}{cc} \left(-0.5\rho -0.15\right){\left(\varepsilon /\varepsilon_{c}\right)}^{3}+\left(3.46-3.8\rho \right){\left(\varepsilon /\varepsilon_{c}\right)}^{2}+(3.6\rho -1.77)\varepsilon /\varepsilon_{c} & \varepsilon \leq \varepsilon_{c}\\ \frac{\varepsilon /\varepsilon_{c}}{\left(2.59-1.5\rho \right){\left(\varepsilon /\varepsilon_{c}\right)}^{2}+\left(2.52-1.4\rho \right)\varepsilon /\varepsilon_{c}+\left(2.9\rho -4.07\right)} & \varepsilon_{c}\leq \varepsilon \end{array}\right.$$

where $\sigma_{c}$ represents peak compression stress for LWMCC; $\varepsilon_{c}$ represents strain corresponding to peak pressure stress; ρ represents mixing ceramsites density, measured in g/cm^3^.

### 4.2. Equation Proofreading

**Figure 8:**
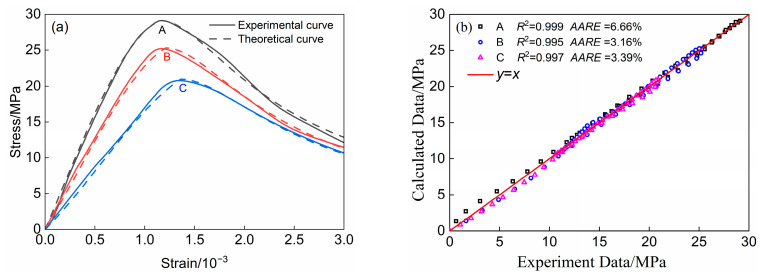
Validation curves of theoretical and experimental values of the modified model. (**a**) Experimental and theoretical curves; (**b**) Calculation accuracy analysis of theoretical curves.

To assess the accuracy of the stress–strain relationship model presented in this study, we compare the theoretical curves derived from Equation (6) with the corresponding experimental curves. The results of this comparison are illustrated in Figure 8a. The model’s accuracy was evaluated using the decidable coefficients and the average relative error, as shown in Figure 8b. The average relative errors for the three groups of specimens—A, B, and C—were 6.66%, 3.16%, and 3.39%, respectively. The new model demonstrates high computational accuracy across all three groups of specimens. This suggests that the new model of the stress–strain relationship for LWMCC, which takes into account the varying densities and ceramic grain content’s impact on the modulus of elasticity and other related parameters, is more reasonable. Consequently, it serves as an effective tool for predicting the axial compressive mechanical properties of LWMCC.

### 4.3. Applicability of Formulas

**Figure 9:**
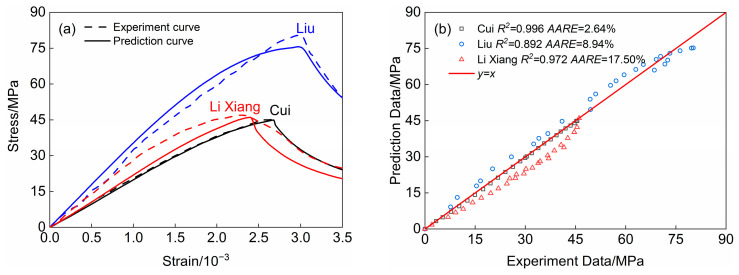
Stress–strain curve comparison. (**a**) Experiment and prediction curves; (**b**) Calculation accuracy analysis of prediction curves.

To validate the applicability of the elastic modulus and stress–strain relationship model formulations proposed in this paper, we compare and analyze the decidability coefficients and average relative errors of our tests against the formulations. This analysis is based on the compression test results of lightweight ceramic concrete conducted by Cui, Liu, and Li Xiang, which are presented in Table 5 and Figure 9. Table 5 indicates that the calculation errors of the elasticity modulus calculation are 0.17, 0.16, and 0.04, respectively. The data in Figure 9 indicates that the various discrete points from the three groups of specimens are clustered around the *y* =*x* curve. The coefficients of determination (*R*^2^) are 0.996, 0.892, and 0.972, respectively, with average relative errors of 2.64%, 8.94%, and 17.50%. This suggests that the elastic modulus and stress–strain relationship model formulations for lightweight ceramic concrete proposed in this paper have a broad range of applicability.

## 5. Conclusions

Through experimental study, this paper investigates the effects of different types and densities of ceramsites, as well as mixed ceramic grains, on the mechanical and deformation properties of LWMCC. By using experimental data, key index features are extracted, leading to the development and validation of a stress–strain relationship model for LWMCC, demonstrating its effectiveness and applicability. The specific conclusions are as follows.

(1) Lightweight ceramic concrete made from a mixture of pebble-type fly ash ceramic particles and crushed shale ceramic particles has better mechanical properties and deformation properties than a single type of lightweight ceramic concrete.
(2) In this paper, the elastic modulus and the stress–strain relationship model of LWMCC are proposed based on density compensation. The prediction errors of the elastic modulus and the stress–strain relationship of the three groups of ceramic concrete are within 7%, and the model demonstrates good accuracy.
(3) Compared with the experimental data from the existing studies both at home and abroad, the error in the elastic modulus and stress–strain relationship predicted by the elastic modulus and stress–strain relationship formula proposed in this paper is within 17%. This indicates that the proposed model formulas can predict the elastic modulus and stress–strain relationship of the LWMCC better and that the elastic-modulus calculation formulas and the stress–strain relationship model proposed in this paper have wide applicability.
(4) Analysis of the compression performance test results indicates the potential of LWMCC to function as a viable structural material in construction applications. In addition, this study presented and validated the elastic modulus formula and the stress–strain relationship formula for LWMCC. Future research will focus on utilizing these formulas to analyze the seismic performance of LWMCC structural elements.

## Figures and Tables

**Table 1:** Properties of ceramsite.

Types of Ceramsite	Grain Size (mm)	Tube Crushing Strength (MPa)	Bulk Density (kg/m^3^)	Coefficient of Water Absorption (%)	Elastic Modulus (×10^4^ MPa)
Shale ceramsite	5~25	3.3	850	9.3	2.3
Fly ash ceramsite	8~20	3.0	750	12.5	1.9

**Table 2:** Mix proportion of LWMCC (kg/m^3^).

Condition	Cement	Sand	Ceramsite		Water
			Shale Ceramsite	Fly Ash Ceramsite	
Specimen A	450	710	350	—	200
Specimen B	450	710	175	200	200
Specimen C	450	710	—	400	200

**Table 3:** Experimental and calculated values of the modulus of elasticity.

Condition	Experiment Value (MPa)	Equation Calculation Value (MPa)	Calculation Error (%)
Specimen A	30,742.5	30,392.6	0.1
Specimen B	23,395.0	23,992.4	2.6
Specimen C	18,496.7	18,164.3	1.8

Where $\delta =\left(X-T\right)/T$, δ represents calculation error; *X* represents equation calculation value; *T* represents experiment value.

**Table 4:** Three types of stress–strain equations.

Source	Calculation Equation		
GB	Ascending segment	$\sigma =\frac{\rho_{c}n}{n-1+x^{n}}E_{c}\varepsilon$	$\rho_{c}=\frac{f_{c}}{E_{c}\varepsilon_{c}},n=\frac{E_{c}\varepsilon_{c}}{E_{c}\varepsilon_{c}-f_{c}},x=\frac{\varepsilon }{\varepsilon_{c}}$
	Descending segment	$\sigma =\frac{\rho_{c}}{\alpha_{c}{\left(x-1\right)}^{2}+x}E_{c}\varepsilon$	
FIP	Ascending segment	$\frac{\sigma }{\sigma_{c}}=-\left[\frac{E_{c}}{E_{c1}}\frac{\varepsilon }{\varepsilon_{c}}-{\left(\frac{\varepsilon }{\varepsilon_{c}}\right)}^{2}\right]/\left[1+\left(\frac{E_{c}}{E_{c1}}-2\right)\frac{\varepsilon }{\varepsilon_{c}}\right]$	
	Descending segment	$\begin{array}{l} \frac{\sigma }{\sigma_{c}}=-\left[\left(\frac{1}{\varepsilon_{c,\lim}/\varepsilon_{c}}\xi -\frac{2}{{\left(\varepsilon_{c,\lim}/\varepsilon_{c}\right)}^{2}}\right){\left(\frac{\varepsilon }{\varepsilon_{c}}\right)}^{2}+\left(\frac{4}{\varepsilon_{c,\lim}/\varepsilon_{c}}-\xi \right)\frac{\varepsilon }{\varepsilon_{c}}\right]\\ \xi =\frac{4\left[{\left(\varepsilon_{c,\lim}/\varepsilon_{c}\right)}^{2}\left(E_{c}/E_{c1}-2\right)+2\left(\varepsilon_{c,\lim}/\varepsilon_{c}\right)-E_{c}/E_{c1}\right]}{{\left[\left(\varepsilon_{c,\lim}/\varepsilon_{c}\right)\left(E_{c}/E_{c1}-2\right)+1\right]}^{2}} \end{array}$	
GZH	Ascending segment	$\begin{array}{l} \sigma /\sigma_{c}=a\left(\varepsilon /\varepsilon_{c}\right)+\left(3-2a\right){\left(\varepsilon /\varepsilon_{c}\right)}^{2}+\left(a-2\right){\left(\varepsilon /\varepsilon_{c}\right)}^{3}\\ a=E_{0}/E_{c1} \end{array}$	
	Descending segment	$\sigma /\sigma_{c}=\left(\varepsilon /\varepsilon_{c}\right)/\left[\alpha {\left(-1+\varepsilon /\varepsilon_{c}\right)}^{2}+\left(\varepsilon /\varepsilon_{c}\right)\right]$	

Where GB represents the Chinese standard GB 50010-2010 [20]; FIP represents the European standard CEB-FIP [21]; GZH represents a book by Z.H. Guo [22]; $E_{c}$ represents the tangential modulus of elasticity; $E_{c1}$ represents the cut-line modulus of elasticity; $E_{0}$ represents the initial modulus of elasticity; $\sigma_{c}$ represents the peak compressive stress of the prism; $\varepsilon_{c}$ represents the peak compressive strain of the prism; $\varepsilon_{c,\lim}$ represents the ultimate compressive strain value; Parameters α are determined according to the concrete strength class and restraint method.

**Table 5:** Data and error analysis of physical and mechanical properties of lightweight ceramic concrete in different studies.

Resource	The Grain Density (kg/m^3^)	Compressive Strength (MPa)	Modulus of Elasticity (GPa)		
			Experimental Value	Equation Calculation Value	Calculation Error
Cui [15]	790	50.56	22.25	26.14	0.17
Liu [2]	860	76.70	32.16	37.31	0.16
Li Xiang [23]	774	51.30	23.62	24.47	0.04

## Data Availability

The data presented in this study are available on request from the corresponding author due to privacy.

## References

[1] Gao S., Huang K., Chu W., Wang W. (2023). Feasibility Study of Pervious Concrete with Ceramsite as Aggregate Considering Mechanical Properties, Permeability, and Durability. Materials.

[2] Liu X., Wu T., Liu Y. (2019). Stress-strain relationship for plain and fibre-reinforced lightweight aggregate concrete. Constr. Build. Mater..

[3] Yu Z., Tang R., Liu G., Guo Z., Huang Q. (2021). Experimental Study on Dynamic Performance of Plain Concrete and Lightweight Aggregate Concrete under Uniaxial Loading. J. Mater. Civ. Eng..

[4] Wang S., Zhao J., Wu X., Yang J., Wang Q. (2023). Elastic Properties and Damage Evolution Analysis for Lightweight Shale Ceramsite Concrete. Int. J. Appl. Mech..

[5] Guo Z.H., Zhang X.Q., Zhang D.C., Wang R.Q. (1982). Experimental study on full stress-strain curves of concrete. J. Build. Struct..

[6] Wang Z.Y., Ding J.T., Guo Y.S. (2005). Stress-Strain curves of structural lightweight aggregate concretes. Concrete..

[7] Ye P., Chen Y., Chen Z., Xu J., Wu H. (2022). Failure Criteria and Constitutive Relationship of Lightweight Aggregate Concrete under Triaxial Compression. Materials.

[8] Liu L.B. (2023). Study on the influence of light aggregate type on the performance of high-performance light aggregate concrete. Transpoworld.

[9] Wu X., Wang S., Yang J., Zhao J., Chang X. (2022). Damage characteristics and constitutive model of lightweight shale ceramsite concrete under static-dynamic loading. Eng. Fract. Mech..

[10] Han B., Xiang T.-Y. (2017). Axial compressive stress-strain relation and Poisson effect of structural lightweight aggregate concrete. Constr. Build. Mater..

[11] Hou D.J., Yan H.D. (2012). Study on the influence of different species and content of superlight ceramsite to the performance of foam concrete. Fujian Constr. Sci. Technol..

[12] Wei H., Liu Y., Wu T., Liu X. (2020). Effect of Aggregate Size on Strength Characteristics of High Strength Lightweight Concrete. Materials.

[13] Yang J.H., Tang Y.L., Yu J.Y., Meng L.F., Cheng Z.X. (2017). Experimental study of cast-in-situ all-lightweight foamed concrete used by shale ceramsite. Ind. Constr..

[14] Overli J.A. (2016). A density-dependent failure criterion for concrete. Constr. Build. Mater..

[15] Cui H.Z., Lo T.Y., Memon S.A., Xing F., Shi X. (2012). Experimental investigation and development of analytical model for pre-peak stress-strain curve of structural lightweight aggregate concrete. Constr. Build. Mater..

[16] (2010). Lightweight Aggregates and Its Test Methods—Part 2: Test Methods for Lightweight Aggregates.

[17] Liu X.B., Li P.J., Ji Y.Q. (2005). The effective elastic modulus of ceramsite and its predication. Concrete.

[18] (2019). Standard for Test Methods of Concrete Physical and Mechanical Properties.

[19] (2019). Technical Standard for Application of Lightweight Aggregate Concrete.

[20] (2010). Code for Design of Concrete Structures.

[21] Fédération Internationale du Béton (2013). fib Model Code for Concrete Structures 2010.

[22] Guo Z.H. (1997). Strength and Deformation of Concrete. Test Basis and Constitutive Relationship.

[23] Li X., Zhang L., Zhao J., Ynag S. (2024). Experimental and numerical simulation study on mechanical properties of ceramsite concrete. Concrete.

